# Fully automated viability and toxicity screening—A reliable all‐in‐one attempt

**DOI:** 10.1002/cam4.7392

**Published:** 2024-06-22

**Authors:** Victoria Liedtke, Romano Weiss, Anastasia Skifov, Stefan Rödiger, Lysann Schenk

**Affiliations:** ^1^ Faculty of Natural Sciences Brandenburg University of Technology Cottbus‐Senftenberg Senftenberg Germany; ^2^ Faculty of Health Sciences Brandenburg University of Technology Cottbus‐Senftenberg Senftenberg Germany

**Keywords:** automated system, bioimage informatics, CRISPR/Cas9, cytotoxicity, viability

## Abstract

**Background:**

The CRISPR/Cas9 technology is nowadays a common tool for genome editing to achieve new insights into, for example, diagnostics and therapeutics in cancer and genetic disorders. Cell proliferation and anticancer drug response studies are widely used to evaluate the impact of editing. However, these assays are often time‐consuming, expensive, and reproducibility is an issue. To overcome this, we developed a fast and cheap assay that combines a fully automated multispectral fluorescence microscopy platform with a nuclei staining and open‐source software analysis.

**Methods:**

Here, we generated different LEDGF/p75 model cell lines to validate the effect on proliferation and chemosensitivity. Therefore, a fast protocol for an optimized all‐in‐one attempt for cytotoxicity screenings and proliferation analysis of adherent cells in a 96‐well plate format was established using differential staining with two fluorescent dyes (Hoechst 33342 and propidium iodide) for live/dead cell discrimination. Subsequently, an automated cell nuclei count and analysis were performed using bioimage informatics.

**Results:**

With the new established assay technology, up to 50,000 cells/well can be detected and analyzed in a 96‐well plate, resulting in a fast and accurate verification of viability and proliferation with consistency of 98% compared to manual counting. Our screening revealed that LEDGF depletion using CRISPR/Cas9 showed a diminished proliferation and chemosensitivity independent of cell line origin. Moreover, LEDGF depletion caused a significant increase in 𝛾H2AX foci, indicating a substantial increase in DNA double strand breaks. LEDGF/p75 overexpression enhanced proliferation and chemoresistance underlining the role of LEDGF in DNA damage response.

**Conclusion:**

Independent of cancer cell type, LEDGF/p75 is a central player in DNA damage repair and is implicated in chemoresistance. Moreover, our automated fluorescence biosensor technology allowed fast and reliable data acquisition without any fixation or additional washing steps. Additionally, data analysis was implemented using the modular open‐source software that can be adapted as needed.

## Graphical abstract

In this manuscript, we provide a fast, easy and cost‐efficient all‐in‐one attempt for analyzing proliferation and cytotoxicity in a wide range of human cancer cell lines. Additionally, it is a reliable, nontoxic, and metabolism‐independent method for comparing genetically modified cell lines or different chemotherapeutic drugs.

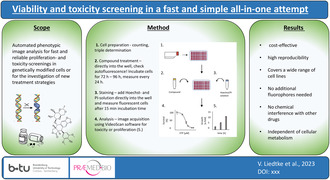

## 1 INTRODUCTION

Genomic alterations can cause cancer, and with the genome editing tool CRISPR/Cas9 (clustered regularly interspaced short palindromic repeats / CRISPR associated enzyme 9), a technology has been developed to explore genetic changes in a fast and easy way.^1^, ^2^ The identification of potential drivers for cancer, as well as potential therapy targets, has been already accelerated through the introduction of RNA interference (RNAi) but CRISPR/Cas9 technology is speeding up the research advances even further. In comparison to other genome editing tools such as transcription activator‐like effector nuclease (TALEN) or zinc finger nucleases, CRISPR/Cas9 is efficient, easy programmable, modifies chromosomal targets with high fidelity. It offers the possibility of multiplex genome editing as well as transcriptional regulation.^3^ Moreover, CRISPR/Cas9 technology allows not only the knockout (KO) of genes and gene modulation but also directed knockin of gene expression cassettes into a safe harbor locus.^4^

A major obstacle in cancer treatment is chemoresistance. The list of involved genes is long and can also vary depending on the type of cancer. The transcriptional co‐activator lens epithelium derived growth factor, LEDGF (also known as dense fine speckled autoantigen of 70 kDa, DFS70), is a multi‐functional chromatin‐binding protein and its longer splice variant LEDGF/p75 is known to be overexpressed in different types of cancer, for example, breast and prostate cancer cells.^5^, ^6^ Moreover, it is crucial for an efficient DNA damage response repair pathway and is involved in chemoresistance.^7^, ^8^, ^9^ During stress conditions, LEDGF/p75 binds to stress‐response elements (STREs) to activate the expression of stress‐related genes, resulting in increased chemoresistance.^6^

A robust drug sensitivity assay is a prerequisite to establish a reliable drug efficacy and potency. Typically, proliferation and cytotoxicity for example, sensitivity towards chemotherapeutics are assessed by colorimetric assays measuring the cell content or metabolic activity. Sulforhodamine B (SRB) stains the cellular protein content and is, since 1990, a widely used and cost‐efficient assay.^10^, ^11^ The binding of the dye is stoichiometric, and its intensity is proportional to the cell mass. Furthermore, the MTT (3‐(4,5‐dimethylthiazol‐2‐yl)‐2,5‐diphenyltetrazolium bromide) assay, which measures mitochondrial NADH dehydrogenase activity, and the ATP assay (which uses, e.g., luciferin to evaluate ATP level), are frequently used metabolic assays. Nevertheless, the reproducibility is often challenging due to many fixing, staining, and washing steps that might cause cell detachment or incomplete excess dye removal. Moreover, most of these assays are time‐consuming. The aforementioned problems that arise by the usage of colorimetric assays can be overcome by the deployment of automatic analysis of image data. In addition, manual assessment of data is prone to error due to the subjectivity of the assessor.

Here we propose a strategy that combines fluorescence staining with bioimage informatics to access cell viability and cytotoxicity in an automated, high‐throughput manner. Using bioimage informatics, extensive image datasets can be analyzed fast and reproducibly.^8^, ^12^ In previous studies, we have described the foundations of a measurement platform which has been used to obtain images for autoimmune diagnostics,^13^, ^14^ DNA damage analysis^12^, ^15^ and biomedical applications^16^ using different biosensor approaches.^17^, ^18^, ^19^

Additionally, CRISPR/Cas9 technology can be used to modulate the expression of oncogenes and its impact on the chemosensitivity. In a previous study, we established already LEDGF‐modified HEp‐2 cell lines which showed an increased chemoresistance with LEDGF/p75 overexpression (LEDGF/p75 OE) and a reversed effect in LEDGF KO cells.^8^ To provide further evidence regarding the changes in chemosensitivity we designed an assay setup to analyze the effect of LEDGF KO and LEDGF/p75 OE in different cell lines.

## 2 MATERIALS AND METHODS

### 2.1 Antibodies

The following antibodies were used in this study: anti‐𝛾H2AX (Cell Signaling Technology Cat# 2577, RRID:AB_2118010, Massachusetts, USA), anti‐C‐LEDGF/p75 (Bethyl‐Laboratories, Cat# A300‐848A, RRID:AB_2171223, Texas, USA), anti‐α‐actinin (Santa Cruz Biotechnology Cat# sc‐17829, RRID:AB_626633, Massachusetts, USA), anti‐β‐tubulin (Cell Signaling Technology, Cat# 2128, RRID:AB_823664, Massachusetts, USA), anti‐mouse‐IgG_κ_ BP‐HRP (Santa Cruz Biotechnology, Cat# sc‐516102, RRID:AB_2687626, Texas, USA), anti‐rabbit HRP (Sigma Aldrich, Cat# AP132P, RRID:AB_90264, Missouri, USA) and α‐Mouse‐IgG‐Alexa Fluor 488 (Abcam Cat# ab150113, RRID:AB_2576208, Cambridge, UK).

### 2.2 Cell lines and culture

Human epithelial type 2 (HEp‐2, (ATCC Cat# CCL‐23, RRID:CVCL_1906), passage number 9–18), human bone osteosarcoma epithelial cells (U2OS, (ATCC Cat# HTB‐96, RRID:CVCL_0042), passage number 5–19) and human colon cell line (LoVo, (ATCC Cat# CCL‐229, RRID:CVCL_0399), passage number 5–16) wild‐type (WT) cells as well as LEDGF KO and EGFP‐LEDGF/p75 overexpressing (OE) cells were grown up to 80% confluence in DMEM/Ham's F12 supplemented with 10% FBS (Biowest, Nuaillé, France), 2 mM l‐glutamine (Merck Millipore, Massachusetts, USA) and penicillin/streptomycin (Merck Millipore, Massachusetts, USA) in a humidified incubator at 37°C and 5% CO_2_.

### 2.3 Cloning

For the generation of LEDGF KO cells, single‐guide (sg) RNA for CRISPR/Cas9‐mediated genome editing sgRNA_DF70_E1 (AGATGAAAGGTTATCCCCAT, targeting exon 1 of *PSIP1* gene) was cloned into the mammalian expression vector pSpCas9 (BB)‐2A‐GFP (PX458; Addgene plasmid # 48138 kindly provided by Feng Zhang, Ph.D.^20^). For the expression of EGFP‐LEDGF/p75 a sgRNA targeting the human safe harbor locus (*AAVS1*, sgRNA_AAVS1: CCAATCCTGTCCCTAG) was cloned into a pSpCas9 (BB)‐2A‐GFP‐plasmid (PX458; Addgene plasmid # 48138), and a gBlock HDR fragment (containing attB sites for Gateway cloning and an EGFP‐LEDGF/p75 coding sequence) into pAAVS1‐P‐CAG‐DEST‐plasmid (Addgene plasmid # 80490, kindly provided by Knut Woltjen, Ph.D.^4^).

### 2.4 Generation of LEDGF‐modified cell clones

CRISPR/Cas9‐modified HEp‐2, U2OS, and LoVo cells were generated according to the method described by Liedtke et al.^8^ Briefly, cells were seeded in six‐well plates (Th. Geyer, Renningen, Germany), incubated for 24 h and subsequently transfected with px458_sgR_DFS70_E1 using Lipofectamine™ 3000 according to manufacturer's instructions (Thermo Fisher Scientific, Massachusetts, USA). For the EGFP‐LEDGF/p75 expression, LEDGF KO cells were co‐transfected with px458_sgRNA_AAVS1 and pAAVS1_CAG‐EGFP‐LEDGF/p75. Transfected cells were enriched by EGFP selection via FACS using S3e cell sorter (Bio‐Rad). Fluorescent, single cell colonies were picked after 7–10 days to establish LEDGF/p75 re‐expressing cell lines. Obtained cell clones were analyzed to verify the integration of the expression cassette at the *AAVS1* locus by PCR as described in Oceguera‐Yanez et al.^4^ showing only heterozygous integrations. The expression level of LEDGF/p75 was verified by western blot analysis due to relatively quantification of protein bands by ImageJ.^21^

### 2.5 Proliferation analysis

Cells were seeded at 5 × 10^3^ cells/well into multiple 96‐well plates (Th.Geyer, Renningen, Germany) and incubated for 24–96 h at 37°C and 5% CO_2_. To determine the accuracy of cell seeding, one plate was used as a 0 h control. For proliferation analysis after 0–96 h, the corresponding plates were stained as explained in Section 2.7 and analyzed as explained in Section 2.8.

### 2.6 Chemotherapeutic treatment

Cells were seeded at 5 × 10^3^ cells/well into 96‐well plates (Th. Geyer, Renningen, Germany) and incubated for 24 h. Subsequently, cells were exposed to 5 μM of etoposide (ETP) (as indicated in the figures and the procedure (Supplement 1—Data S1)).

### 2.7 Staining with DNA incorporating dyes

After treatment for the indicated time (Figures 3 and 4), cells were stained with Hoechst 33342 (4 μg/mL, Merck Millipore, Massachusetts, USA) and PI (2 μg/mL AppliChem, Darmstadt, Germany) in 1×PBS (Biowest, Nuaillé, France) for 15 min at 37°C, 5% CO_2_ and images were taken by the automated VideoScan imaging platform^13^, ^22^ with UPlan S Apo objective 10×/ 0.16∞/−/FN 26.5 (Olympus, Hamburg, Germany) using an Olympus IX fluorescence microscope (Olympus, Hamburg, Germany).

### 2.8 Digital image analysis and bioimage informatics

Analysis of proliferation and cytotoxicity assays after Hoechst/PI staining was performed using the fully automated multispectral fluorescence microscopy VideoScan platform^13^, ^22^ In principle, and for users who do not possess the VideoScan system, our all‐in‐one attempt is compatible with common fluorescence microscopes. PI/Hoechst‐stained cells were measured using an exposure time of 0.5–1 s. Each well of the 96‐well plate was dissected into 12 subunits, imaged in meandering sequence at 10× magnification and reassembled as a single image. Images were subsequently analyzed using bioimage informatics as follows. All image processing steps were performed using the library scikit‐image v.0.17.2. The obtained images were converted to grayscale and all parts of the image outside the analyzed well were masked using the image average to not interfere with detection. Additionally, the image was slightly smoothed before detection using median filtering (4 × 4 neighborhood; skimage.filters.median) to reduce the noise. Cell count per image was determined via Laplacian of Gaussian Blob detection as implemented in scikit‐image skimage.feature.blob_log; v.0.17.2; following settings: min_sigma: 1.5, max_sigma: 4, num_sigma: 10, threshold: 0.005, overlap: 0.35, exclude_borders: False. To exclude dead cells, the PI‐positive cells were subtracted from the number of Hoechst‐positive cells. Prominent problems during image acquisition and data analysis were addressed in a table in (Supplement 1 and 2—Data S1).

### 2.9 Sulforhodamine B assay

To determine cell proliferation, cells were seeded at a density of 5 × 10^3^ cells/well in a 96‐well plate (Th. Geyer, Renningen, Germany) and a SRB assay was performed according to nature protocols.^11^ Briefly, 96‐well plates were fixed with 10% ice cold TCA solution for 1 h at 4°C, followed by washing 4× with slow‐running tap water. Plates were allowed to air dry and 0.057% SRB solution was added directly into the wells and incubated a RT for 30 min. Plates were rinsed again 4× with tap water containing 1% acetic acid and allowed to air dry at room temperature. Tris‐base solution (10 mM, pH 10.5) was added directly into the wells and incubated on a shaker for 5 min to solubilize the protein‐bound SRB‐dye. Measuring was performed by OD analysis at 510 nm.

### 2.10 Immunofluorescence

Cells were seeded at 5 × 10^3^ cells/well on 10‐well slides (Hecht Assistant, Sondheim v. d. Rhön, Germany) and incubated for 24 h. For analysis, cells were fixed with 4% formaldehyde for 15 min at RT and permeabilized with 0.3% Triton X‐100 (AppliChem, Darmstadt, Germany) while blocking with 5% BSA/PBS. Primary antibody was added and incubated at RT for 1 h. Slides were washed with 1×PBS, then incubated with secondary antibody and DAPI (5 μg/mL) for 1 h in the dark at RT. Fluorophore photostability was increased by coating slides with mounting medium (Roti®‐Mount FluorCare, Carl Roth GmbH, Karlsruhe, Germany). Analysis was performed using a confocal laser scanning microscope LSM 800 (Zeiss, Oberkochen, Germany), 60× magnification with oil immersion (microscope settings are listed in Supplement 3—Data S1). Foci formation (100–250 nuclei/image) was analyzed using NucDetect software, excluding mitotic cells (NucDetect 0.15.0, written in Python 3.9, available at https://pypi.org/project/NucDetect/).

### 2.11 Immunoblotting

Cells (1 × 10^6^) were harvested and resuspended in 50 μL 2× Lämmli‐buffer and protein content was determined using Pierce™ BCA Protein Assay Kit (Thermo Fisher Scientific, Massachusetts, USA). Immunoblotting was performed using standard protocols.^23^ Anti‐C‐LEDGF was diluted in 2% milk powder (Carl Roth, Karlsruhe, Germany) in TBS/0.1% Tween‐20 while anti‐α‐actinin and anti‐β‐tubulin antibodies were diluted in 5% BSA (Carl Roth, Karlsruhe, Germany) in TBS/0.1% Tween‐20 (AppliChem, Darmstadt, Germany). HRP‐conjugated secondary antibodies were diluted in 5% milk powder in TBS/0.1% Tween‐20. Non‐saturated and background‐subtracted band intensities were quantified using ImageJ software.^22^ LEDGF protein levels were normalized to loading control. Prior anti‐α‐actinin incubation, PVDF membrane was stripped with stripping buffer (2% SDS, 500 mM Tris/HCl (pH 6.8), 0.8% β‐mercaptoethanol) for 30 min at 50°C.

### 2.12 Statistical analysis

All data were statistically analyzed with the statistical computing language *R* ≥ 4.2.1.^24^ The Kolmogorov–Smirnov test was used for testing normal distribution. To control the α error inflation, the Bonferroni correction is applied or Tukey's HSD test (stats::TukeyHSD()) is used to test the differences between the mean values of the sample for significance. Thereby, the stats::TukeyHSD() function uses the Tukey–Kramer procedure^25^ *p* < 0.05‐ were considered as significant. Experiments were conducted with at least three biological replicates. Data were further analyzed using *RKWard* v. 0.7.5^26^ for the *R* statistical computing environment using the packages *visreg* v. 2.7.0^27^ and *report* v. 0.3.5. A linear model was estimated using ordinary least squares (OLS). The foci count data were processed using the *countfitteR* ^28^ package to determine the distributions and mean values. Data were further analyzed using RKWard v. 0.7.1z + 0.7.2 + devel2^13^ for the R statistical computing environment. Dose–response curves were fitted (95% confidence interval) with multiparametric functions (EXD3: Three‐parameter exponential decay model; LL4: Four‐parameter log‐logistic model) from the drc package.^29^ The optimal model was selected by using the Akaike information criterion (AIC) as criterion.

## 3 RESULTS

### 3.1 Software enables reliable quantitative cell counting with life‐death discrimination for cytotoxicity screenings

**FIGURE 1:**
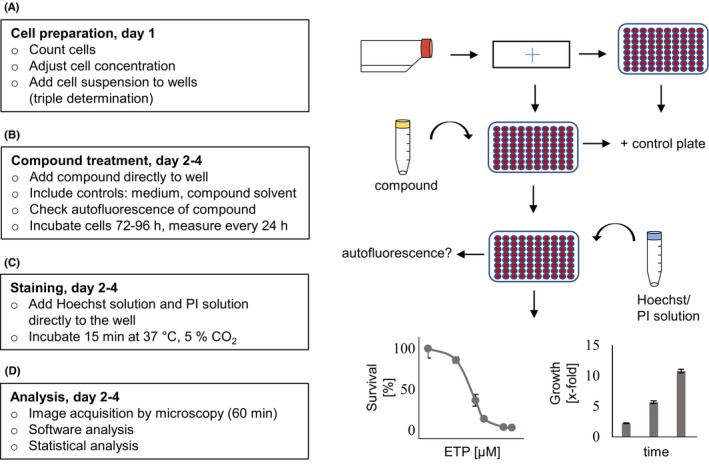
Workflow for automated fluorescent‐based proliferation and cytotoxicity screening. (A) After counting, cells are seeded at appropriate cell densities into 96‐well plates. (B) Compound of interest is added directly to the cells without washing. Include compound solvent and medium only control. Check possible autofluorescence of the compounds ahead of the experiment! (C) After treatment, Hoechst/PI‐solution is directly added to the cells in the well. If background fluorescence is detectable, a washing step with 1x PBS is required. (D) Pictures of each well are taken by a fully automated imaging platform (e.g., VideoScan), followed by digital image analysis (by e.g., VideoScan analyzer). Determination of growth and, if applicable, ED_50_ is determined by data analysis and statistical analysis.

So far, we have used the SRB assay for proliferation and chemosensitivity analysis, but this assay is time‐consuming and requires several washing steps. Moreover, a fixation step is needed, which can cause dislodging of adherent cells. To achieve a good reproducibility, a higher number of replicates was often necessary. Therefore, a fast fluorescence‐based approach using Hoechst/PI staining of non‐fixed, living cells in combination with an automated microscopy platform was established (Figure 1).

**FIGURE 2:**
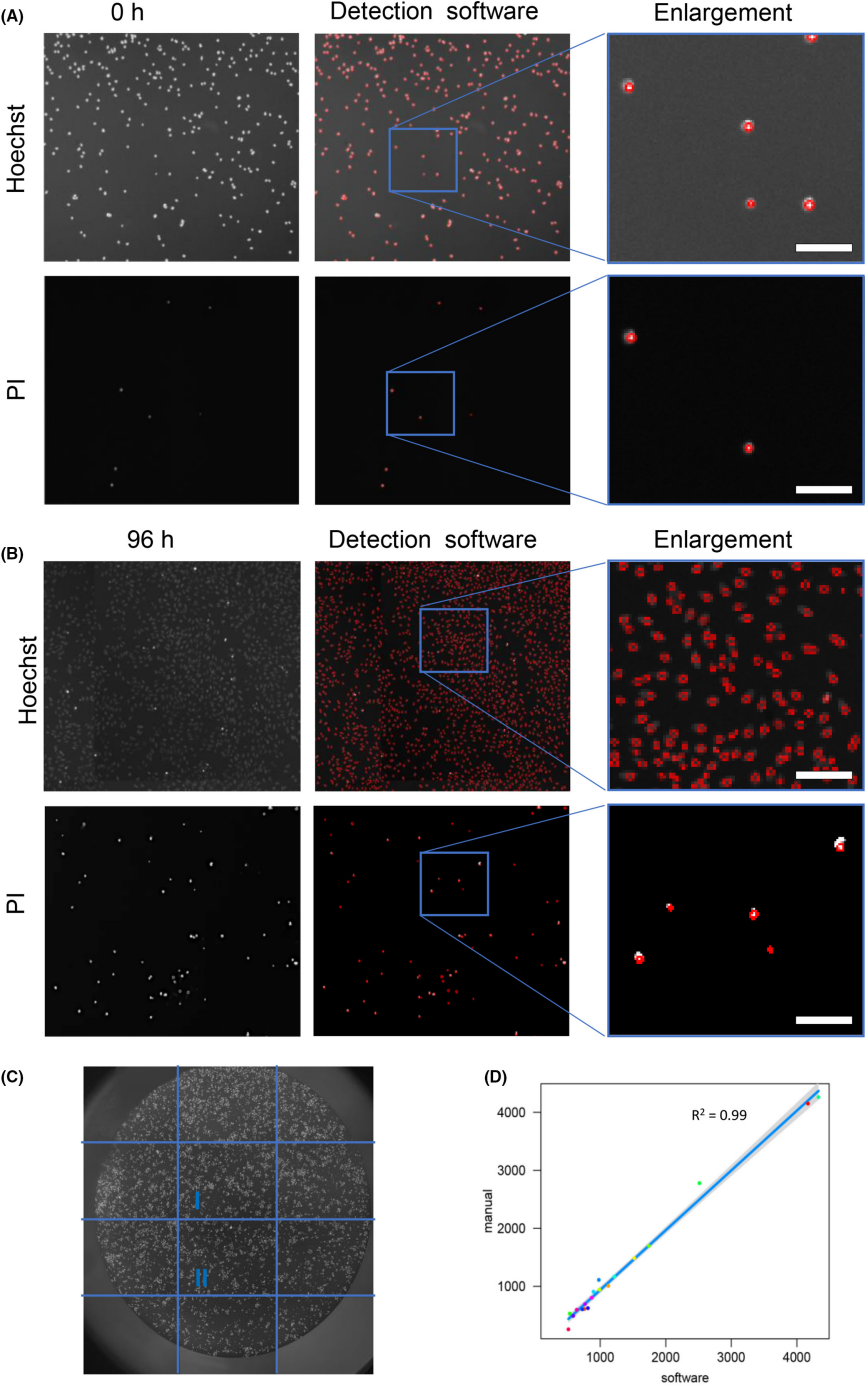
Detection of stained nuclei by digital image analysis software and comparison of cells counted manually and by digital image analysis software. (A) HEp‐2 cells stained with Hoechst and counterstained with PI directly after seeding (0 h) and (B) after 96 h of growth. Images were taken by a fully automated microscopy platform, nuclei are visible as light gray spots and can be overlayed. Digital image analysis software uses Laplacian‐of‐Gaussian Blob detection, as implemented by the skimage library. Each detected nucleus is marked with a red circle. To determine cell viability, cells stained with PI are subtracted from the total number of Hoechst‐positive cells. Scale bar = 100 μm. (C) Segmentation of an image taken from one well of a 96‐well plate. Inner subareas (I and II) were used for comparison between manual and automated analysis. (D) An amount of 20 inner subareas (indicated by colored dots) of a 96‐well plate cultured with Hoechst‐stained HEp‐2 cells were analyzed in 10× magnification. The amount of manually (manual) counted HEp‐2 cells was plotted versus the number of cells counted by digital image analysis software (software).

The Hoechst/PI staining allows a cell count (rather than protein content or metabolic activity) as well as live/dead discrimination. As representative shown in Figure 2 for HEp‐2 cells, cells should be evenly distributed (Figure 2A), and stained properly with Hoechst and PI to allow single cell detection (Figure 2A,B) even after 96 h. We applied a Python script^30^ for automatic cell counting. The enlarged image sections show the precision of the software, which was tested on U2OS, HEp‐2, and LoVo cells. Detection limits can be considered below 500 and above 50,000 cells per 96 well, depending on cell morphology, due to insufficient focus points or clumpy cell formation.

To verify the accuracy of automated detection, approximately 25,000 DAPI‐stained cells were also manually counted. To ensure accuracy during manual counting, the image of the well was divided into subareas (Figure 2C). Representative subareas were analyzed and counts are shown in Figure 2D. Thereby, both counts (manually counted: 25,125 cells, detected by software: 25,693 cells) differ 2% from each other (Figure 2) and can be considered as comparable methods. We fitted a linear model (estimated using OLS) to predict manual counting with software counting (formula: manual ~ software). The model explains a statistically significant and substantial proportion of variance (*R* ^2^ = 0.99, *F*(1, 17) = 2042.69, *p* < 0.001, adj. *R* ^2^ = 0.99). The model's intercept, corresponding to software = 0, is at −91.45 (95% CI [−175.24, −7.66], *t*(17) = −2.30, *p* = 0.034). Within this model, the effect of software is statistically significant and positive (beta = 1.03, 95% CI [0.98, 1.08], *t*(17) = 45.20, *p* < 0.001; Std. beta = 1.00, 95% CI [0.95, 1.04]). Standardized parameters were obtained by fitting the model on a standardized version of the dataset.

### 3.2 Automated image analysis reveals: LEDGF/p75 expression affects proliferation and chemoresistance in a range of cell lines

**FIGURE 3:**
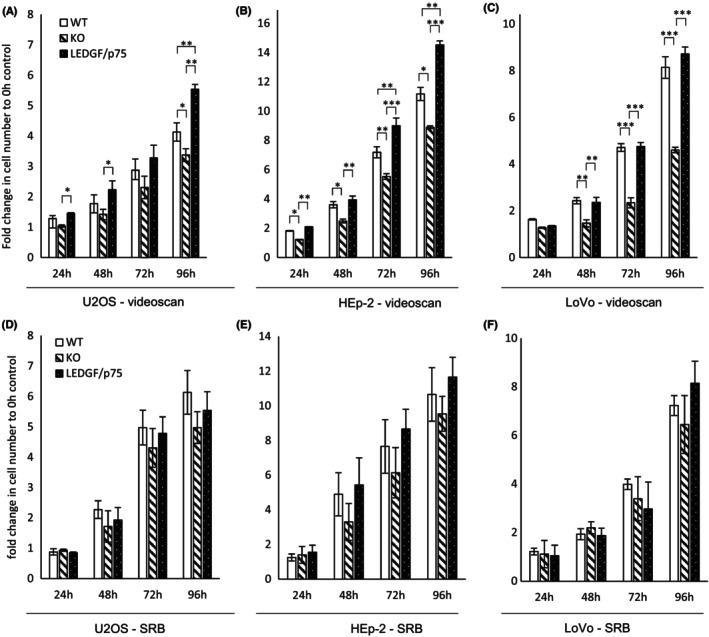
LEDGF/p75 expression levels affect proliferation. Wildtype (WT), LEDGF knockout (KO), and LEDGF/p75 (OE) cells were seeded, cultivated for indicated time periods and then co‐stained with Hoechst and PI (A–C, the amount of living cells was determined by subtracting PI‐positive cells from Hoechst‐positive cells.) or analyzed by the SRB assay (D–F). Proliferation of non‐treated (A) U2OS cells for 24 h, (p_WT‐KO_ = 0.0632, p_KO‐OE_ = 0.0134, p_OE‐WT_ = 0.4341), 48 h (p_WT‐KO_ = 0.2707, p_KO‐OE_ = 0.0397, p_OE‐WT_ = 0.3434), 72 h (p_WT‐KO_ = 0.6396, p_KO‐OE_ = 0.0990, p_OE‐WT_ = 0.5716) and 96 h (p_WT‐KO_ = 0.0400, p_KO‐OE_ = 0.0021, p_OE‐WT_ = 0.0022) (B) HEp‐2 cells for 24 h (p_WT‐KO_ = 0.0196, p_KO‐OE_ = 0.0014, p_OE‐WT_ = 0.0770), 48 h (p_WT‐KO_ = 0.0687, p_KO‐OE_ = 0.0249, p_OE‐WT_ = 0.6958), 72 h (p_WT‐KO_ = 0.0040, p_KO‐OE_ = 0.00007, p_OE‐WT_ = 0.0025) and 96 h (p_WT‐KO_ = 0.0389, p_KO‐OE_ = 0.0007, p_OE‐WT_ = 0.0151) and (C) LoVo cells for 24 h, 48 h (p_WT‐KO_ = 0.0052, p_KO‐OE_ = 0.0076, p_OE‐WT_ = 0.9277), 72 h (p_WT‐KO_ = 0.0008, p_KO‐OE_ = 0.0007, p_OE‐WT_ = 0.9901) and 96 h (p_WT‐KO_ = 0.0005, p_KO‐OE_ = 0.0002, p_OE‐WT_ = 0.4588) analyzed by digital image analysis software. Proliferation of non‐treated (D) U2OS cells, (E). HEp‐2 cells and (F). LoVo cells analyzed by the SRB assay. * = *p* > 0.05, ** = *p* > 0.01, *** = *p* > 0.001.

After accuracy verification of the software, we examined genetically LEDGF‐modified U2OS, HEp‐2, and LoVo cell lines regarding their proliferation capacity and chemosensitivity. Here, initially, we compared the SRB and PI/Hoechst staining assays with each other. In comparison to the automated image analysis software, SRB assays showed no significant difference in growth behavior (Figure 3), if only three technical replicates are used.

**FIGURE 4:**
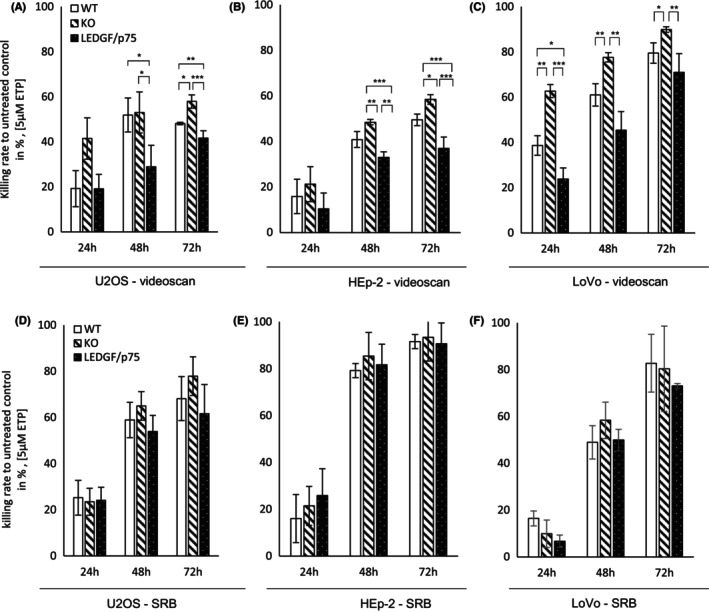
LEDGF/p75 expression affects chemoresistance towards etoposide. Wildtype (WT), LEDGF knockout (KO), and LEDGF/p75 (OE) cells were seeded and after 24 h, treated with 5 μM etoposide (ETP) for indicated the times and subsequently co‐stained with Hoechst and PI (A–C, the amount of living cells was determined by subtracting PI‐positive cells from Hoechst‐positive cells) or analyzed by the SRB assay (D–F). After 24 h growth, killing of (A) U2OS cell lines treated with 5 μM etoposide for 24 h (p_WT‐KO_ = 0.0699, p_KO‐OE_ = 0.0688, p_OE‐WT_ = 0.9991), 48 h (p_WT‐KO_ = 0.9880, p_KO‐OE_ = 0.0392, p_OE‐WT_ = 0.0469) and 72 h (p_WT‐KO_ = 0.0194, p_KO‐OE_ = 0.0002, p_OE‐WT_ = 0.0035), (B) HEp‐2 cell lines treated with 5 μM etoposide for 24 h (p_WT‐KO_ = 0.8391, p_KO‐OE_ = 0.7181, p_OE‐WT_ = 0.9762), 48 h (p_WT‐KO_ = 0.0091, p_KO‐OE_ = 0.0002, p_OE‐WT_ = 0.0072) and 72 h (p_WT‐KO_ = 0.0430, p_KO‐OE_ = 0.0006, p_OE‐WT_ = 0.0099) and (C) LoVo cell lines treated with 5 μM etoposide for 24 h (p_WT‐KO_ = 0.0025, p_KO‐OE_ = 0.0002, p_OE‐WT_ = 0.0306), 48 h (p_WT‐KO_ = 0.0625, p_KO‐p75_ = 0.0035, p_OE‐WT_ = 0.0841) and 72 h (p_WT‐KO_ = 0.2205, p_KO‐OE_ = 0.0303, p_OE‐WT_ = 0.3204) were analyzed by digital image analysis software. Additionally, after 24 h growth, killing of (D) U2OS cells, (E) HEp‐2 cells and (F). LoVo cells treated with 5 μM etoposide for indicated time points were analyzed by the SRB assay. Survival rate was determined in comparison to the untreated control. * = *p* > 0.05, ** = *p* > 0.01, *** = *p* > 0.001.

In the VideoScan analysis, the investigated WT cell lines exhibited different proliferative capacities while U2OS showed only a threefold increase in cell number after 72 h, LoVo and HEp‐2 cells showed a fivefold and sevenfold increase, respectively (Figure 3A). LEDGF KO significantly reduced the proliferation rate in faster proliferating LoVo and HEp‐2 cells, while in U2OS cells only after longer cultivation (96 h) the proliferation in LEDGF KO cells stagnated in comparison to the WT. As expected, the proliferation analysis showed that LEDGF/p75 overexpression cells are proliferating significantly faster than LEDGF KO cells, correlating with longer cultivation time independent of the cell type; however, this effect was less pronounced in slower dividing U2OS cells (Figure 3). LEDGF OE in HEp‐2 cells was strong also in comparison to the WT expression level (Figure 6D) which was reflected by a significantly enhanced proliferation in comparison to the WT (Figure 3B). In addition, the chemosensitivity towards etoposide regarding the LEDGF status was investigated (Figure 4). As there was no difference in growth behavior between untreated cells and cells treated with DMSO, untreated cells were used as control for calculation. A low etoposide concentration (5 μM) already resulted in 40%–60% killing rate after 48 h in all WT cells. Similar to the proliferative behavior, LEDGF KO caused a significant increase in the killing rate as compared to the WT cells; however in U2OS, cells this effect was diminished and only after 72 h etoposide visible. This enhanced chemosensitivity was effectively reversed by overexpressing LEDGF/p75. Moreover, LEDGF OE resulted partially, even, in a significantly increased chemoresistance. As already shown in the proliferation study, the SRB assay was able to detect increasing killing rates in all cell lines (Figure 4D–F), but no significant difference dependent on the LEDGF expression level.

### 3.3 Knockout of LEDGF/p75 renders cells more chemosensitive towards etoposide

**FIGURE 5:**
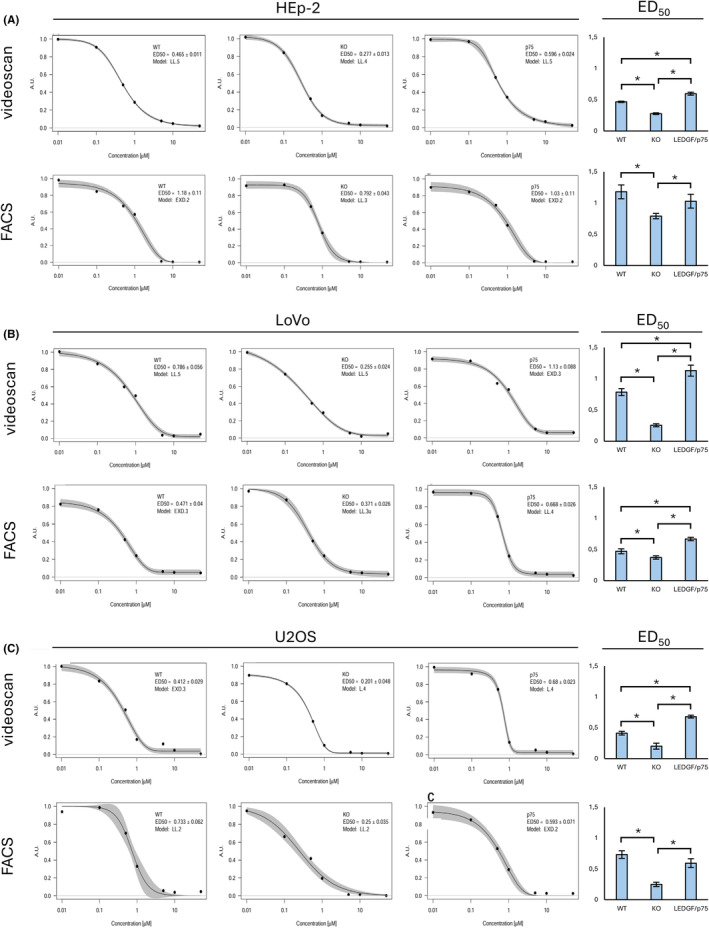
ED_50_ determination of different cell lines using FACS analysis and automated image analysis software. Determination of ED_50_ value was performed on wildtype (WT), LEDGF knockout (KO), and LEDGF/p75 (OE) cells, treated with indicated concentrations of etoposide for 48 h etoposide, followed by 3 days of recovery. Viable (A) HEp‐2 cells, (B) LoVo‐cells, and (C) U2OS cells were analyzed by the digital image analysis software (upper histograms) whereas a parallel run was analyzed by FACS (lower histogram). Dose–response curves were fitted with multiparametric functions from the drc package.^29^, ^31^ The optimal model was selected by using the AIC as criterion. The triangles on the x‐axis represent the dose of etoposide needed to reach ED_50_. *p < 0.05.

The median effective dose (ED_50_) was determined to draw conclusions about the efficacy of etoposide as a chemotherapeutic agent in LEDGF KO or LEDGF/p75 knockin mutations in various reference and colorectal cancer cell lines. While FACS analysis showed much higher overall ED_50_ values in HEp‐2 cells and no difference between HEp‐2 WT and HEp‐2 LEDGF/p75 OE cells, automated image analysis software acted more sensitive and detected lower overall ED_50_ valued and a significant difference in resistance when comparing HEp‐2 LEDGF/p75 OE cells with HEp‐2 WT cells (Figure 5A). The analysis of the LoVo cell lines showed that the ED_50_ values for all three cell lines are very close to each other and that significant but weak differences can be observed, whereas the analysis using automated image analysis shows a well separated and significantly different ED_50_ distribution between the WT, the LEDGF KO and the LEDGF/p75 overexpressing cell lines (Figure 5B). Looking at the analysis of the ED_50_ values for the U2OS cells, it is noticeable that the FACS analysis does not show any significant differences between U2OS WT and U2OS LEDGF/p75. The automated image analysis software can clearly show this small but significant difference (Figure 5C).

### 3.4 Generation of different CRISPR/Cas9‐modified LEDGF cancer cell models

**FIGURE 6:**
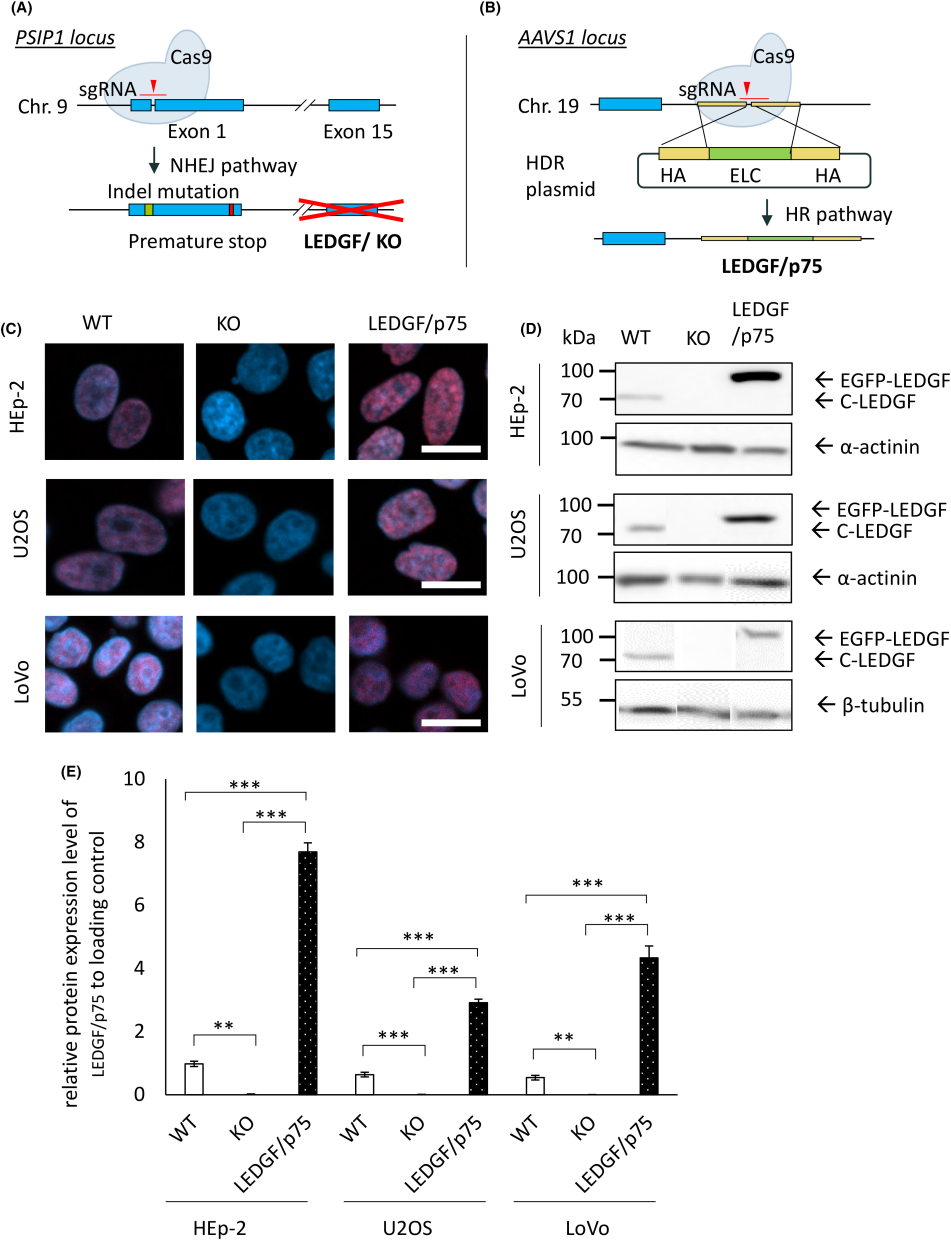
Generation of CRISPR/Cas9‐modified LEDGF cancer cell models. (A) Scheme of LEDGF knockout generation: Specific sgRNA for Exon 1 of LEDGF‐coding gene (*PSIP1*) was designed to introduce a target‐specific DNA double‐strand break (DNA DSB) with CRISPR/Cas9 technology. Due to endogenous error‐prone non‐homologous end joining (NHEJ) pathway, indel mutations can cause a frameshift leading to premature stop codons and subsequent gene knockout (LEDGF KO). (B) Scheme of LEDGF/p75 knockin generation: LEDGF overexpressing cells were created by introducing a DNA DSB at genomic safe harbor locus *AAVS1* using an *AAVS1*‐specific sgRNA and an HDR template containing EGFP‐LEDGF expression cassette (ELC) flanked by homologous arms (HA) to the cutting site at the *AAVS1* locus. After the induction of a DSB, homology‐directed repair (HR) pathway mediates the integration of the ELC. (C) Indirect immunofluorescence of HEp‐2, U2OS and LoVo cell lines: wildtype (WT), LEDGF KO (KO) and LEDGF/p75 overexpression (OE). Representative confocal images show nuclei (DAPI, blue) and C‐LEDGF (red, C‐terminal binding antibody) (scale bar = 20 μm). (D) Immunoblot using antibodies against C‐terminal LEDGF and α‐actinin or β‐tubulin as loading control. (E) Relative expression level of LEDGF/p75 in WT, LEDGF KO and LEDGF/p75 OE in HEp‐2 (p_WT‐KO_ = 0.0022, p_KO‐p75_ = 1 × 10^−7^, p_WT‐p75_ = 3 × 10^−7^), U2OS (p_WT‐KO_ = 0.0003, p_KO‐p75_ = 1 × 10^−7^, p_WT‐p75_ = 6 × 10^−7^) and LoVo (p_WT‐KO_ = 0.0432, p_KO‐p75_ = 1.7 × 10^−6^, p_WT‐p75_ = 3.8 × 10^−6^) cell lines, compared to the corresponding loading control. **p < 0.01, ***p < 0.001.

The generation of CRISPR/Cas9‐modified LEDGF Hep‐2 cell models has been described previously.^8^ Additionally, LEDGF‐modified U2OS and LoVo cell lines were generated. To achieve a complete LEDGF KO, a specific sgRNA targeting exon 1 of *PSIP1* gene was designed (Figure 6A). Thereby, the transient transfection with a non‐viral px458_DFS70_E1 vector allowed an enrichment of Cas9‐positive cells via FACS sorting using EGFP as a marker. Subsequently, LEDGF/p75 expression cassette was integrated at the human safe harbor locus *AAVS1* (directed integration to avoid nonspecific side effects) using CRISPR/Cas9 (Figure 6B). Cell lines were established from single‐cell clones and complete LEDGF KO was verified on protein level (Figure 6C,D) and on genomic level (Supplement 4—Data S1). The robust EGFP‐tagged LEDGF/p75 expression and correct subcellular localization (in the nucleus) was determined by immunofluorescence (Figure 6C) and immunoblotting (Figure 6D), where LEDGF/p75 OE cells show an up to 10‐fold increased LEDGF/p75 expression, compared to the expression in WT cells (Figure 6E). Note, the membrane was stripped between LEDGF/p75 and α‐actinin protein detection and to allow an appropriate identification of EGFP‐LEDGF/p75 expression, an anti‐rabbit secondary antibody was used, whereas an anti‐mouse antibody was used for the detection of α‐actin expression. In LoVo cell lines, an anti‐β‐tubulin antibody was used due to low detection level (Figure 6D).

### 3.5 LEDGF KO causes an increase in DNA damage

**FIGURE 7:**
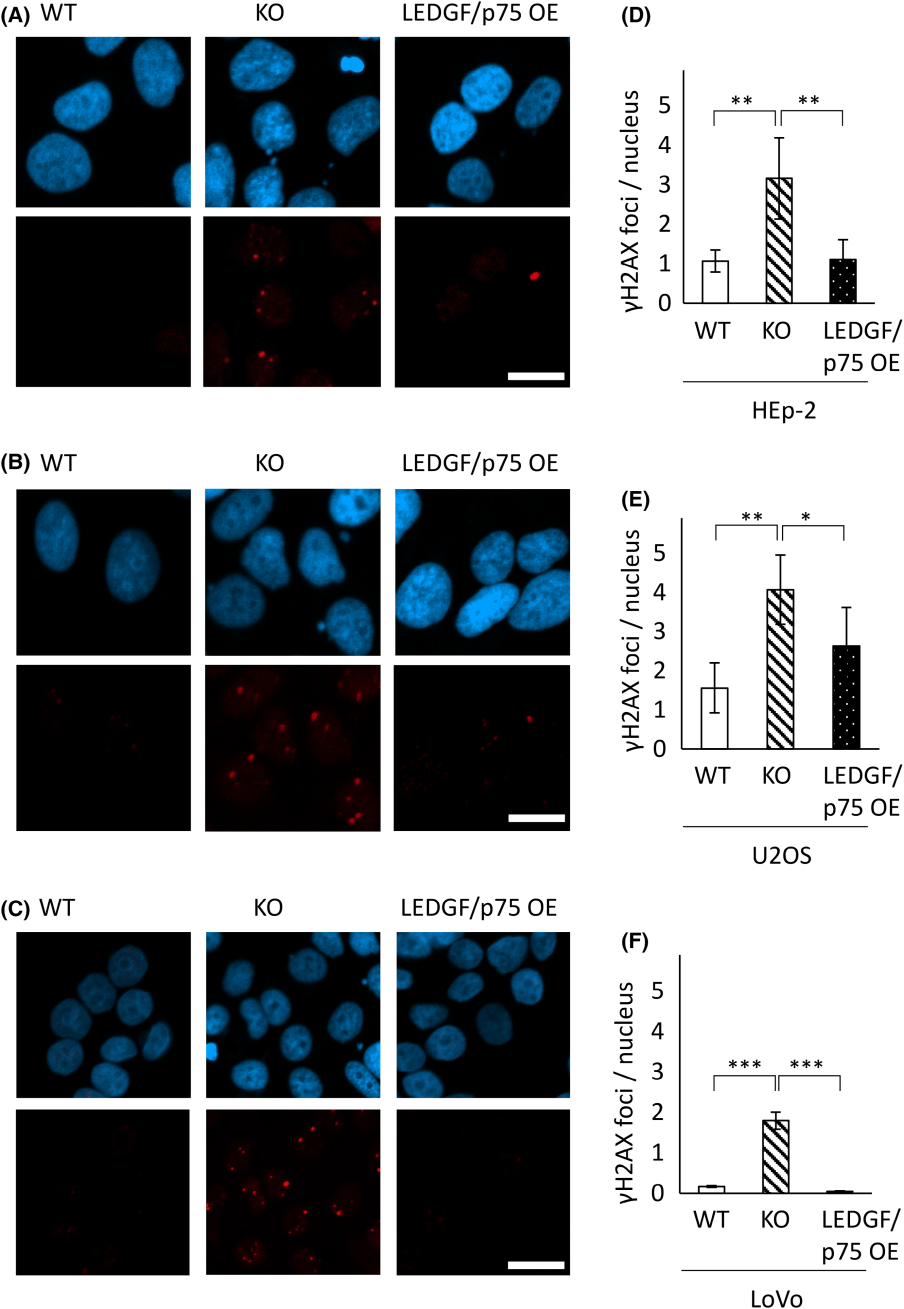
LEDGF/p75 re‐expression reverses dysregulation of DNA damage response. Representative confocal images after indirect immunofluorescence with DAPI (blue) and anti‐𝛾H2AX (red) of (A) HEp‐2 WT, KO, and LEDGF/p75 OE cells (B) U2OS WT, KO, and LEDGF/p75 OE cells and (C) LoVo WT, KO, and LEDGF/p75 OE cells. Confocal images were then used for the analysis of 𝛾H2AX foci per nucleus (at least 100 nuclei were counted) in (D) HEp‐2 WT, KO, and LEDGF/p75 OE cells (p_WT‐KO_ = 0.005, p_KO‐p75_ = 0.002, p_WT‐p75_ = 0.997), (E) U2OS WT, KO, and LEDGF/p75 OE cells (p_WT‐KO_ = 0.002, p_KO‐p75_ = 0.044, p_WT‐p75_ = 0.163) and (F) LoVo WT, KO, and LEDGF/p75 OE cells (p_WT‐KO_ = 8.5 × 10^−6^, p_KO‐p75_ = 5.3 × 10^−6^, p_WT‐p75_ = 0.469) using the NucDetect software, *n* = 3 (Scale bar = 20 μm): *p < 0.05, **p < 0.01, ***p < 0.001.

LEDGF is involved in DNA damage repair, and we have previously shown that upon LEDGF KO, the 𝛾H2AX foci formation is markedly increased, even without chemotherapeutic treatment.^8^ 𝛾H2AX is the marker for DNA double‐strand breaks (DSB), the most severe DNA lesion. Therefore, we determined the 𝛾H2AX foci formation in the investigated cell models. The immunofluorescence analysis (Figure 7A–C) revealed significant upregulated expression of 𝛾H2AX in all LEDGF KO cells compared to the corresponding WT cells in HEp‐2 (3‐fold, p_WT‐KO_ = 0.005), U2OS (2.6‐fold, p_WT‐KO_ = 0.002) and LoVo (10‐fold, p_WT‐KO_ = 8.5 × 10^−6^) cells. This effect can be significantly reversed by LEDGF/p75 overexpression (Figure 7D–F).

## 4 DISCUSSION

Proliferation analysis and cytotoxicity screenings are standard procedures in medical research. The demand on the tests is that they are reliable, fast, and easy to handle. Plenty of methods are available, but many of them have drawbacks. The routine use of colorimetric staining assays like NRU assay,^32^ crystal violet assay,^33^, ^34^ and SRB assay^11^ (Supplement 5—Data S1) often leads to inconclusive results with high standard deviations, caused by excess binding of dyes, incomplete washing steps and a low sensitivity. In addition, these assays stain any proteins that are present. Alternatively, metabolic activity assays, such as the MTT/MTS, XTT, LDH, WST assays^35^, ^36^, ^37^, ^38^, ^39^ or fluorometric assays like the almarBlue assay,^40^, ^41^ the CFDA‐AM assay,^40^, ^42^ and the Annexin V assay^43^ can be used (Supplement 5—Data S1). These are also colorimetric assays, but detection depends on the conversion of added chemicals which correlates with the amount of mitochondria in a given cell and activity of cellular metabolism. Therefore, these assays cannot be used for comparison between different cell lines. Nevertheless, metabolic assays show an increased cytotoxicity, chemicals may interfere with possible tested drugs and again no quantitative statement can be made about the corresponding cell number. Another accurate possibility for analysis of cytotoxicity can be the analysis of exposed phosphatidylserine residues on the cellular membrane using the Annexin‐V assay. During apoptosis, phosphatidylserine flips from the inner cell membrane to the outer cell membrane, and the amount corresponds to early or late apoptosis. Due to fluorescence labeling, these events can be tracked by a flow cytometer or fluorescence microscope, allowing the detailed analysis of each single cells^44^ (Supplement 5—Data S1). Nevertheless, all protocols contain many individual washing and staining steps which lead to multiplying errors. Moreover, many assays are not only time‐consuming but also cost intensive. To tackle this problem, we propose a fast and cheap protocol for a straightforward all‐in‐one staining and detection procedure (protocol in Supplement 1—Data S1). Our assay requires no washing step, is independent of metabolic activity or amount of mitochondria, no additional fluorophore structures need to be added. There is no need to worry about interference with chemical substances, Hoechst/PI solution is directly added to living adherent cells with a high sensitivity of at least 500 cells/well of a 96‐well plate and subsequently stained nuclei are detected by automated microscopy and images are processed by software packages for viable cell quantification. In principle, and for users who do not possess the VideoScan system, our all‐in‐one attempt is compatible with all common fluorescence microscopes with and without automatic stage. After the detection procedure cells can even be used for further analysis like indirect immunofluorescence analysis. To verify the accuracy of our software, we performed a comparison between our program and manually counted cells, which resulted in a consistency of almost 98% of counted single cells (Figure 2). Regarding the commercially available CCK‐8 assay, which is one of the most used assays for cell proliferation and cytotoxicity,^45^, ^46^, ^47^ our assay is 42 times less expensive (CCK‐8 kit: 0.67 €/1 reaction, our assay: 0.016 €/1 reaction), more sensitive—we only need half of the cells to exceed the detection limit and not limited by metabolic activity of the cell. Our approach can make semi‐quantitative statements, such as colorimetric assays, but can also accurately quantify the total number of cells and the number of dead cells simultaneously. Other protocols use fixed cells^48^ for the analysis of DNA bound dyes while we use living cells where Hoechst stains all nuclei, but PI incorporates only in late apoptotic or necrotic cells whose cell membrane is permeable. This procedure can serve for a wide range of experiments like multiplex testing of different drugs to calculate ED_50_ level of different compounds, analyzing the increase/decrease in cytotoxicity after genomic modulation of cells (e.g., after knock‐out or knock in of certain genes). It can be also used for checking proliferation of cells prior to experiments or comparing proliferation of different cell lines with potential new anticancer drugs.

In our study, the newly established assay was used to pursue the investigation of CRISPR/Cas9‐generated LEDGF KO and LEDGF/p75 overexpressing cell lines and their chemosensitivity. LEDGF/p75 has been reported to be overexpressed in different tumor and cancer cell lines^7^, ^49^ and to promote repair of DSBs by the homologous recombination repair pathway.^9^ In our previous studies, we underlined the crucial role of LEDGF as a main player in initiating DNA repair processes.^8^ The lack of LEDGF expression resulted in an accumulation of 𝛾H2AX,^8^ an early responder of the DNA repair and marker for DSBs.^50^ In the present study, 𝛾H2AX was significantly increased (depending on cell line between 2.6‐ and 10.5‐fold, Figure 6) in LEDGF KO cells independent of the cell line, whereas LEDGF/p75 OE was able to rescue the WT phenotype with only background 𝛾H2AX foci. Interestingly, 𝛾H2AX is also an indicator for genome integrity and persistent 𝛾H2AX foci formation is also related to tumorigenesis and genome instability.^51^, ^52^ This may suggest that LEDGF participates in the maintenance of genome integrity. Moreover, depletion of SETD2, the only methyltransferase catalyzes the tri‐methylation of histone‐h3 lysine‐36 (H3K36me3), also causes persistent 𝛾H2AX foci.^52^, ^53^ The histone reader LEDGF preferentially binds to H3K36me3 and its chromatin binding is reduced upon SETD2 depletion.^9^ Additionally, our group found out, that persistent 𝛾H2AX foci relate to insufficient degradation of 𝛾H2AX due to interaction of LEDGF/p75 with the nuclear proteasome activator PA28𝛾.^8^ The increased 𝛾H2AX foci correlated with decreased proliferation rates in all LEDGF KO cells. This effect was reversed upon LEDGF overexpression and for strong LEDGF OE, the proliferation rate was even more elevated than in the WT cells. In breast cancer, upregulation of LEDGF/p75 expression also resulted in increased proliferation rates.^54^

Additionally, chemosensitivity towards etoposide depending on the LEDGF status was investigated. As expected, LEDGF KO induced a sensitizing effect, which indicates that increased 𝛾H2AX is related to genomic instability and the cells are less stress resistant. On the contrary, LEDGF/p75 OE not only reversed this sensitizing effect, but partially led to an even higher chemoresistance against etoposide. Previously, chemoresistance has been described to be correlated with increased LEDGF/p75 expression in prostate cancer.^6^ Furthermore, the higher chemoresistance of LEDGF/p75 OE might also be related to the role of the shorter splice variant LEDGF/p52, which plays rather a pro‐apoptotic role and is considered as a counterpart of antiapoptotic LEDGF/p75.^55^ In fact, WT cell lines most likely express both splice variants LEDGF/p75 and LEDGF/p52. However, we only verified the expression of the longer splice variant p75 (Figure 5D). Nonetheless, LEDGF sgRNA targets exon 1 and thereby knocks out both splice variants. LEDGF/p75 OE model was established using LEDGF KO cells and therefore potential LEDGF/p52 expression was not reconstituted. Therefore, we cannot exclude that LEDGF/p52 might counteract LEDGF/p75 in WT cells leading to an altered proliferation and chemosensitivity. Nonetheless, specific depletion of LEDGF/p52 had no effect on chemosensitivity towards camptothecin.^9^

## 5 CONCLUSION

In summary, our data revealed that LEDGF depletion causes increased DNA damage as indicated by elevated 𝛾H2AX and resulting most likely in genome instability. Chemosensitivity towards etoposide depends on the expression level of LEDGF/p75, and higher LEDGF/p75 expression levels lead to chemoresistance. Moreover, we established a fast and cheap high‐throughput assay for proliferation and cytotoxicity screenings. This fluorescence‐based approach uses Hoechst/PI staining on non‐fixed, living cells in combination with automated microscopy and analysis, leading to accurate cell count with live/dead discrimination. For the analysis, we used open‐source software that can be used modularly in different scenarios. Moreover, all needed components are cheap, no cell fixation or washing is required.

## AUTHOR CONTRIBUTIONS

**Victoria Liedtke:** Conceptualization (equal); data curation (equal); formal analysis (equal); funding acquisition (equal); investigation (equal); methodology (equal); project administration (equal); visualization (equal); writing – original draft (equal). **Romano Weiss:** Data curation (equal); formal analysis (equal); investigation (equal); software (equal); writing – review and editing (equal). **Anastasia Skifov:** Investigation (equal). **Stefan Rödiger:** Conceptualization (equal); funding acquisition (equal); methodology (equal); resources (equal); supervision (equal); validation (equal); writing – review and editing (equal). **Lysann Schenk:** Conceptualization (equal); funding acquisition (equal); methodology (equal); resources (equal); supervision (equal); validation (equal); writing – review and editing (equal).

## FUNDING INFORMATION

This work was supported by PRAEMED.BIO—Präzisionsmedizin durch biomarkerbasierte Diagnostik of the Federal Ministry of Education and Research (03WKDB2C, BMBF, Germany) and the Friedrich‐Naumann Foundation for Freedom.

## CONFLICT OF INTEREST STATEMENT

The authors have no conflicts of interest to declare.

## ETHICS STATEMENT

Not Applicable.

## CLINICAL TRIAL REGISTRATION NUMBER

Not Applicable.

## Supporting information


Data S1:

## Data Availability

The data that support the findings of this study are available from the corresponding author upon reasonable request.
