# Supplementary material for: Fully automated viability and toxicity screening—A reliable all‐in‐one attempt
Source: Cancer Med. 2024 Jun 22;13(12):e7392. doi: 10.1002/cam4.7392 (PMC11193055; doi:10.1002/cam4.7392)
Supplement: Supplementary file 1 — Data S1: [file CAM4-13-e7392-s001.docx]

**Supplement**

**Supplement 1: Protocol for fully automated proliferation analysis and toxicity screening (including ED_50_ determination).**

- - - 1. **Materials**
- Adherent cell line of interest (HEp-2, U2OS, LoVo)
- Appropriate cell culture medium (DMEM/Ham’s F12)
- 200 mM L-glutamine (Merck Millipore, Massachusetts, USA)
- Penicillin/streptomycin (50 µg/ml) (Merck Millipore, Massachusetts, USA)
- Fetal bovine serum (Biowest, Nuaillé, France)
- 2.5 % (w/v) trypsin/EDTA (0.05 % / 0.02 % in PBS w/o Ca^2+^ / Mg^2+^) (Merck Millipore, Massachusetts, USA)
- 0.5 % trypan blue in 0.9 % NaCl (Carl Roth, Karlsruhe, Germany)
- Dimethyl sulfoxide (DMSO) (Carl Roth, Karlsruhe, Germany)
- Chemotherapeutic of interest e.g., etoposide (Sigma Aldrich, Missouri, USA)
- 4 µg/ml Hoechst solution in double-distilled H_2_O (ddH_2_O)
- 2 µg/ml propidium iodide (PI) solution
  - - 1. **Equipment**
- 96-well clear flat-bottom polystyrene tissue-culture plates (Th. Geyer)
- T75 tissue culture flasks or 100 mm tissue culture plates (TPP)
- Inverse microscope
- Automated imaging platform, e.g., VideoScan platform (13,14) with UPlan S Apo objective 4x/ 0.16 ∞/-/FN 26.5 (Olympus, Hamburg, Germany)
- Olympus IX fluorescence microscope (Olympus, Hamburg, Germany)
- Multichannel pipette
  - - 1. **Procedure**

**3.1 Cells for plating (1 h)**

3 days before starting the experiment, grow appropriate cells in T75 cell culture flask. At the day of plating, cells should be in a good vital state, and the confluence of maintenance culture should be at 60-70 % (Suppl. 2 A). Cell distribution in the 96-well plate needs to be homogenous, to avoid clump formation (Suppl. 2 B).

**3.2 Preparation of treatment** **(1 h)**

Working solutions of chemotherapeutic treatments should be freshly prepared. For the preparation of a 100 µM etoposide solution, dilute etoposide stock-solution (100 mM in DMSO) 1/1000 in 1 x growth medium and keep on ice until use. This working stock is then further diluted (serial dilutions are recommended to limit pipetting errors) to the needed concentrations.

→ Critical step: for the ED50-determination, different concentrations of compound are needed. Remember to prepare serial dilutions (2-5 fold) in a wide concentration range (e.g., 0.1 µM to 10 µM etoposide).

For obtaining an optimal and reliable result, samples should be prepared in three replicates. Additionally, controls like medium control and DMSO control (depending on the solvent used for drugs) are required.

The concentration range of the treatment, which is needed to determine the half maximal effective dose (EC_50_), should follow some considerations:

- - - - 1. Use the effective dose known for the cell line, if not known, use a wide range of concentrations for initial experiments.
        2. Three different concentrations are the minimum, but to lower the standard deviation, 5-7 different concentrations are recommended.
        3. The untreated control represents 0 % mortality, whereas the positive control leads to 100 % mortality.
        4. The achieved mortality rate should range between 20-90 %.

Depending on the treatment duration (24 - 72 h) multiple 96-well plates need to be prepared. Additionally, an untreated, 0 h control plate is needed.

**3.3 Cell preparation (3 – 4 h preparation, 24 h incubation)**

1. On day 1, plate cells in 96-well plates. Therefore, remove the culture medium from the culture flask (cells should be in logarithmic growth phase) and wash the cell monolayer once with 1 x sterile PBS. In this protocol, CRISPR/Cas9-modified HEp-2, U2OS and LoVo cell lines were used. Generation of the used cell lines is described in (8).

2. Add enough 1x trypsin / EDTA to cover the cell monolayer (e.g., 1 ml trypsin / EDTA required for a T75 cell culture flask). Incubate the cells in 1 x trypsin / EDTA until they attach at 37 °C, 5 % CO_2_. When the cells start to round up and detach, add 1 volume of FBS-containing culture medium (e.g., add 1 ml FBS-containing medium to the previous 1 ml trypsin / EDTA) to inactivate the trypsin and to disperse cells from the culture flask. Mix thoroughly to obtain a homogenous cell suspension.

3. Transfer the cell suspension to a sterile polypropylene tube. Dilute a small aliquot of cell suspension in a 1:1 dilution with 0.5 % trypan blue solution. Determine the cell concentration by counting the cells using a hemocytometer chamber under a microscope. Do not proceed with the assay, if a large amount of your cells are dead or clumpy

4. Adjust the cell concentration with growth medium to obtain an appropriate seeding density, make sure to thoroughly mix cell suspension before diluting them. For a 96-well plate, we recommend a seeding density of 5×10^4^ cells/ml with osteosarcoma cell line U2OS, laryngeal cancer cell line HEp-2 and colorectal cancer cell line LoVo. Calculate the amount of needed wells depending on the concentration range, replicates (at least triplicate) and controls plus 10-20 % excess volume of cell suspension.

5. Add 100 µL cell suspension per well of a 96-well plate.

6. Set aside a plate containing only cell suspension without treatment as your non-treated control.

→ Critical step: This control plate will be stained after 4 h to identify possible seeding errors. For the 0 h control plate, please continue with step 10.

7. Incubate the plates at 37 °C in a humidified chamber with 5 % CO_2_ for 24 h before proceeding with compound treatment.

**3.4 Compound treatment (1.5 h preparation, up to 72 h incubation)**

8. Prepare the final working solution by using the 100 µM working stock of etoposide. The final working concentrations need to be 2 x concentrated, as 100 µL per well is added straight to the well containing already 100 µL medium. For HEp-2 cells, final concentrations of 0.1 µM, 0.5 µM, 1 µM, 5 µM and 10 µM as well as an only medium control (non-treated) are used. Remember to prepare 10-20 % excess volume per dilution.

9. Add 100 µL of the 2x concentrated etoposide working solution **without** removing the 100 µL culture medium in the 96-well plates.

10. Incubate the plates at 37 °C in a humidified chamber with 5 % CO_2_ and measure the cell number every 24 h (up to 72 h) and analyze as described below.

→ Critical: check the auto-fluorescence of your used compounds. If your compound has an auto-fluorescence, a washing step before staining is necessary.

**3.5 Staining (1 h )**

11. Prepare a Hoechst/PI working solution by diluting the stock solutions in 1x growth medium to a final concentration of 4 µg/µL for Hoechst and 2 µg/µL for PI.

12. Without removing the cell culture supernatant, gently add 20 µL of Hoechst/ PI solution and incubate the plates for 20 min at 37 °C and 5 % CO_2_ in a humidified chamber.

13. Before measuring, check the staining of the cells is uniform throughout the wells using a fluorescence microscope.

**3.6 Analysis (1.5 h image acquisition, 3 h image analysis)**

14. Place the plate in the microscope and adapt the exposure times for Hoechst and PI. In our experiments, Hoechst staining requires an exposure of 1sec and PI 0.5sec at a 10 x magnification.

→ Critical step: Before measuring, always check one or more wells manually. If the exposure time is too high, cells cannot be distinguished in the analysis. If exposure is too weak, cells cannot be detected.

16. For each well, a picture of every well for Hoechst and PI are separately composed. Whereby, cells detected with Hoechst represent the total number of cells in the well. Counterstaining with PI allows the detection of apoptotic and dead cells. Apoptotic and dead cells will be subtracted from the total number of cells to determine the number of viable cells per well (Fig. 2). In our experiments, image acquisition took 1.5 h for a complete 96-well plate in which the cells were stained with DAPI and PI.

17. Using the video scan analyzer script for Python 3.7 (23), each well of the 96-well plate is analyzed for its total number of cells (Hoechst incorporation) and number of apoptotic/dead cells (PI incorporation). In our experiments, image analysis was performed on an Intel® Core™ i7-8550U CPU with 1.80 GHz and a NVIDIA GeForce150 graphics card PC and required 3 h. The results are then summarized in an Excel table. The results are then summarized in an Excel table.

18. Calculate the cell viability and toxicity using the formulae below.

$$change= \frac{{(value}_{cell line A}- {value}_{cell line B})}{{value}_{cell line B}}$$

Whereas the value of cell line A (e.g., HEp-2 LEDGF KO) at a specific time point describes the change in growth compared to cell line B (e.g., HEp-2 WT).

The percentage of cell killing after chemotherapeutic treatment was calculated the following:

$$viability= \frac{sample}{control} \cdot100 \%$$

$$killing=100 \%-viability$$

Thereby, the “sample” represents the value of living cells at a specific time point treated with a chemotherapeutic substance (e.g., treatment with 5 µM etoposide for 24 h) compared to the non-treated control.

**3.7 Immunofluorescent staining (2 h preparation, 26 h incubation)**

19. To explain differences in growth rate and toxicity, an immunofluorescence (in this experiment of rabbit anti-𝛾H2AX) analysis can be performed. Therefore, seed cells at 5×10^3^ cells/well on 10-well slides (Hecht Assistant, Sondheim v. d. Rhön, Germany) and incubate for 24 h.

20. For analysis, fix cells with 4 % formaldehyde for 15 min at RT and permeabilize with 0.3 % Triton X-100 (AppliChem, Darmstadt, Germany) while blocking with 5 % bovine serum albumin in PBA (BSA/PBS).

21. Add primary antibody and incubate at RT for 1 h. Wash slides with 1 x PBS, then incubate with secondary antibody and DAPI (1:500, 2.5 mg/mL) for 1 h in the dark at RT.

22. Increase fluorophore photostability by coating slides with mounting medium (Roti®-Mount FluorCare, Carl Roth GmbH, Karlsruhe, Germany).

23. Perform analysis using a confocal laser scanning microscope LSM 800 (Zeiss, Oberkochen, Germany).

24. Foci formation (250-500 nuclei/image) can be analyzed using NucDetect software, excluding mitotic cells (NucDetect 0.15.00, written in Python 3.7, available at <https://pypi.org/project/NucDetect/>).

**3.8 Troubleshooting**

| **Problem** | **Reason** | **Solution** |
| --- | --- | --- |
| Software is not counting the cells | Non-optimal seeding can lead to clumpy cells  (Suppl. 2 B) | Before starting the analysis, check the cell distribution. Cells should look healthy and should be equally distributed. |
| Cells cannot be focussed | Dust and dirt may interfere with automatic focus detection (Suppl. 2 C) | Clean the surface with an ant-dust tissue and repeat analysis. |
| Software detects artifacts on the edges of single pictures | Non-optimal thinning leads to border detection (Suppl. 2 D) | Thinning must be optimized and checked before analysis. |
| Wells are permanently overexposed | Some treatments (e.g., Irinotecan) have auto-fluorescence properties (Suppl. 2 E) | Test the autofluorescence of your used drugs before starting the experiment. Wells with autofluorescent drugs need additional washing steps before microscopic analysis |

**Supplement 2:**

**
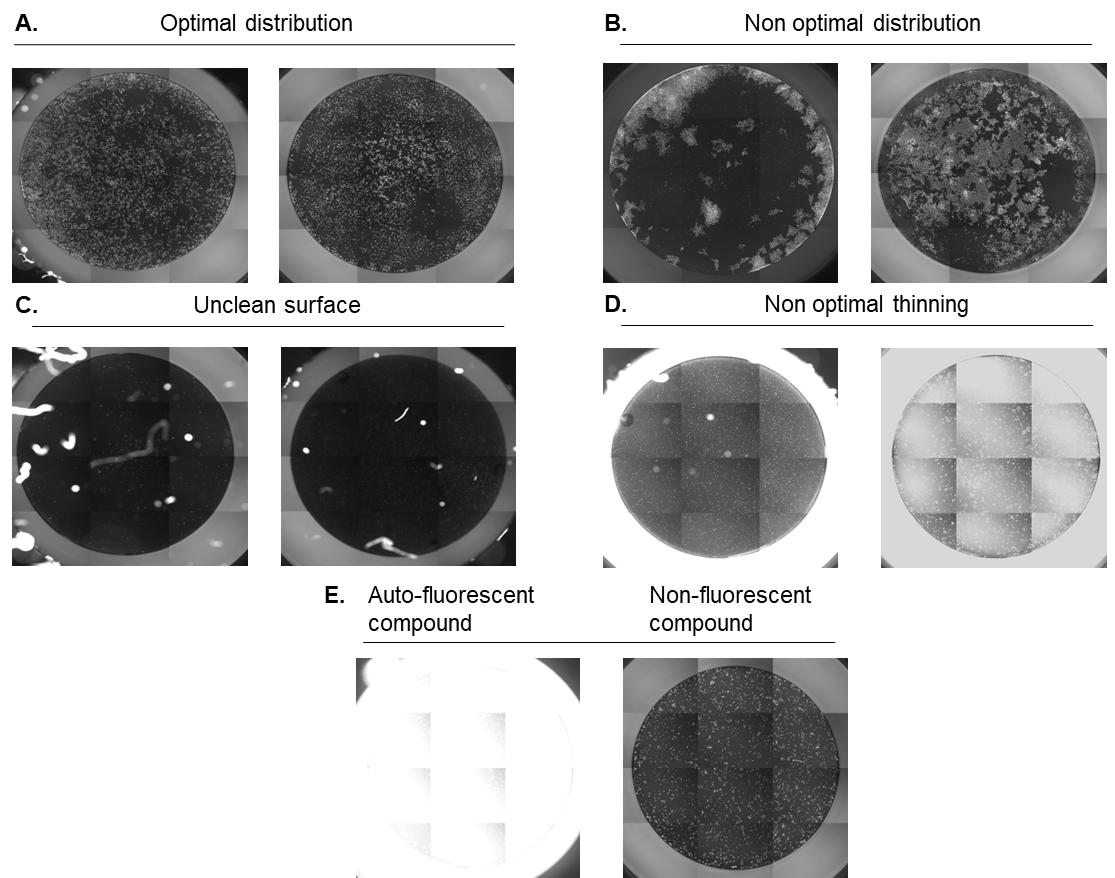
Supplement 2: Potential problems for image analyzing software. A.** Optimal seeding conditions. **B**. Non-optimal seeding conditions lead to clumpy cell growth and multiple cell layers, where single nuclei cannot be discriminated by e.g. VdeoScan analyzer software anymore. **C.** Small dust pieces need to be removed from the plate by cleaning with an anti-dust tissue, persistent dust leads to wrong focus and undetectable nuclei. **D.** Thinning must be adjusted for optimal nuclei detection and to avoid border detection. **E.** Compounds with auto-fluorescen may interfere with different fluorescent channels and lead to undetectable nuclei.

**Supplement 3:**

| **Setting** | **DAPI** | **𝛾H2AX** |
| --- | --- | --- |
| *Laser* | 405 nm | 640 nm |
| *Laser Strength* | 0.25 % | 0.55 % |
| *Exposure* | automatic | |
| *Pinhole* | 50 µm | |
| *Master Gain* | 657 V | 780 V |
| *Digital Offset* | 0 | -17 |
| *Digital Gain* | 1.0 | |

**Supplement 3:** Microscope settings used at the confocal laser scanning microscope LSM 800 (Zeiss, Oberkochen, Germany).

**Supplement 4:**

| **Clone** | **Cell line** | **Selection marker** | **Allele-specific mutations** | **Premature stop codon (pos. in CDS)** |
| --- | --- | --- | --- | --- |
| **PSIP1 KO** | Hep-2 | EGFP (Cas9 activity) | 32 nt del (at pos. 37-68)  11 nt del (at pos. 47-57) | aa 19 (exon 2)  aa 26 (exon 2) |
| **PSIP1 KO** | U2OS | EGFP (Cas9 activity) | 188 nt deletion (at pos. 117 – 305)  8 nt ins. (at pos 107) + 196nt del. (at pos. 108-304) | loss of exon 1  loss of exon 1 |
| **PSIP1 KO** | LoVo | EGFP (Cas9 activity) | 45 nt deletion (at pos. 200 – 244)  45 nt deletion (at pos. 200 – 244) | aa 551 (non-LEDGF coding from aa 7) |

**Supplement 4: Sequencing analysis of *PSIP1* exon 1 knockout cell lines. A.** Sequencing analysis of used LEDGF KO clones showed indel mutation, which resulted in a premature stop codon.

**Supplement 5: Comparison of different proliferation assay**

| **Assay type** | **Assay description** | **Time** | **Advantages** | **Disadvantages** | **Linearity** | **Sensitivity** | **Costs** |
| --- | --- | --- | --- | --- | --- | --- | --- |
| Automatized fluorescence imaging | VideoScan with motorized stage | 1.5–2 h | •Accurate and reproducible  •All-in-one-attempt  •No additional washing steps  •automatized fluorescence microscope with motorized stage guarantees consistent quality | •Inaccurately measured wells can only be measured again at the end of the process | 0.99 (Fig. 4D) | •Detection limit: 5×10^2^ cells / 96-well | Middle |
|  | VideoScan with manual stage | 3-5 h | •Correction of each well in real time | •Fluorescence microscope with manual stage  •Low reproducibility due to user variation  •Time consuming | 0.99 (Fig. 4D) | •Detection limit: 5×10^2^ cells / 96-well | Middle |
| Colorimetric assays | SRB-assay (11) | 2–4 h | •Easy to use | •High standard deviation caused by excess / too less binding of dye  •No discrimination between live and dead cells  •Tricholoacetic acid needed to fix cells  •inability to account for variation in cellular metabolism throughout the life cycle | 0.61 – 0.98 | •Detection limit: 1×10^3^ cells /96-well | Low |
|  | NRU-assay (32) | 3-4 h | •Easy to use | •Possible precipitation of NRU-dye  •No absolute cell numbers  •inability to account for variation in cellular metabolism throughout the life cycle | 0.99 | •Detction limit:  1×10^4^ cell / 96-well | Low |
|  | Crystal-Violet Assay (33) | 2–3 h | •Easy to use | •inability to account for variation in cellular metabolism throughout the life cycle | 0.88 - 0.98 | •Detection limit:  16 ng protein | Low |
| Metabolic assays | Tetrazolium reduction assays  (MTT / MTS-, XTT-, WST-assay) (35, 36) | 4–6 h | •Easy to use  • | •MTT insoluble in water à organic solvents necessary to solubilize crystals  •MTT is cytotoxic  •No differentiation between cell death and growth inhibition  •Depending on metabolic activity  •overestimate the efficacy of drugs that alter cell metabolism | 0.92 – 0.99 | •Detection limit: 1×10^3^ cells / 96-well | Low |
|  | LHD-assay  (35, 36, 56) | 1–1.5 h | •Multiple time point analysis in one assay | •High background readings (LDH)  •Interference with culture compounds | 0.98 - 0.99 | •Detection limit:  5×10^3^ cells / 96-well | Low |
| Fluorimetric assay | Apoptosis assay (41) | 2–3 h | •High sensitivity, detection of early phase apoptosis | •Expensive reagents  •Susceptible to false positive results | N/A | •Detection of single cells | High |
|  | almarBlue-Assay (37) | 6-8 h | •Non-toxic | •Depending on metabolic activity  •Possible interference from test compounds | 0.99 | •Detection limit:  50 cells / 96-well | Low |
|  | CFDA-AM-assay (37) |  | •Non-toxic | •Depending on metabolic activity  •Possible interference from test compounds | 0.97 | N/A | Low |
